# Correction: Saqib et al. Antidiarrheal and Cardio-Depressant Effects of *Himalaiella heteromalla* (D.Don) Raab-Straube: In Vitro, In Vivo, and In Silico Studies. *Plants* 2022, *11*, 78

**DOI:** 10.3390/plants13141999

**Published:** 2024-07-22

**Authors:** Fatima Saqib, Faisal Usman, Shehneela Malik, Naheed Bano, Najm Ur-Rahman, Muhammad Riaz, Romina Alina Marc (Vlaic), Crina Carmen Mureşan

**Affiliations:** 1 Faculty of Pharmacy, Bahauddin Zakariya University, Multan 60000, Pakistan; faisal.usman@bzu.edu.pk (F.U.); shehneelamalik1@gmail.com (S.M.); 2 Faculty of Veterinary and Animal Sciences, MNS-University of Agriculture, Multan 60000, Pakistan; bnaheed61@gmail.com; 3 Department of Pharmacy, Shaheed Benazir Bhutto University, Sheringal 18050, Pakistan; najm@sbbu.edu.pk (N.U.-R.); pharmariaz@gmail.com (M.R.); 4 Food Engineering Department, Faculty of Food Science and Technology, University of Agricultural Sciences and Veterinary Medicine, 400372 Cluj-Napoca, Romania; crina.muresan@usamvcluj.ro

There was an error in the original publication [1]. A correction has been made to the Discussion section. The last paragraph of the Discussion section has been removed along with references 33–38 because they are not related to the text. References 33–38 have also been removed.

The deleted paragraph reads as follows:

“The pretreatment of various chemical, physical, physicochemical, and biological methods have been suggested to improve enzymatic hydrolysis; these techniques can be improved by the activities of extract [33–35]. In addition, the pretreatment and pyrolysis process require a high amount of external heat for (1) drying the washed biomass, (2) biomass torrefaction, and (3) biomass pyrolysis. Biomass drying and pyrolysis require a high amount of external heat for drying and torrefaction [36,37]. The plant also contains potassium (K), calcium (Ca), sodium (Na), and magnesium (Mg), which will significantly affect the behaviors of extract activity. It is essential to remove the metals by adopting different methods [38].”

The deleted references are the following:

“33. Zhao, C.; Cao, Y.; Ma, Z.; Shao, Q. Optimization of liquid ammonia pretreatment conditions for maximizing sugar release from giant reed (*Arundo donax* L.). *Biomass Bioenergy* **2017**, *98*, 61–69.

34. Qiao, X.; Zhao, C.; Shao, Q.; Hassan, M. Structural Characterization of Corn Stover Lignin after Hydrogen Peroxide Presoaking Prior to Ammonia Fiber Expansion Pretreatment. *Energy Fuels* **2018**, *32*, 6022–6030.

35. Chen, D.; Cen, K.; Cao, X.; Chen, F.; Zhang, J.; Zhou, J. Insight into a new phenolic-leaching pretreatment on bamboo pyrolysis: Release characteristics of pyrolytic volatiles, upgradation of three phase products, migration of elements, and energy yield. *Renew. Sustain. Energy Rev.* **2021**, *136*, 110444.

36. Chen, D.; Cen, K.; Cao, X.; Zhang, J.; Chen, F.; Zhou, J. Upgrading of bio-oil via solar pyrolysis of the biomass pretreated with aqueous phase bio-oil washing, solar drying, and solar torrefaction. *Bioresour. Technol.* **2020**, *305*, 123130.

37. Chen, D.; Chen, F.; Cen, K.; Cao, X.; Zhang, J.; Zhou, J. Upgrading rice husk via oxidative torrefaction: Characterization of solid, liquid, gaseous products and a comparison with non-oxidative torrefaction. *Fuel* **2020**, *275*, 117936.

38. Chen, D.; Cen, K.; Chen, F.; Ma, Z.; Zhou, J.; Li, M. Are the typical organic components in biomass pyrolyzed bio-oil available for leaching of alkali and alkaline earth metallic species (AAEMs) from biomass? *Fuel* **2020**, *260*, 116347.”

Due to this correction, the order of some references has been adjusted accordingly. 

The authors state that the scientific conclusions are unaffected. This correction was approved by the Academic Editor. The original publication has also been updated.
